# Integration of genomics, transcriptomics and metabolomics identifies candidate loci underlying fruit weight in loquat

**DOI:** 10.1093/hr/uhac037

**Published:** 2022-02-07

**Authors:** Ze Peng, Chongbin Zhao, Shuqing Li, Yihan Guo, Hongxia Xu, Guibing Hu, Zongli Liu, Xiuping Chen, Junwei Chen, Shunquan Lin, Wenbing Su, Xianghui Yang

**Affiliations:** 1 State Key Laboratory for Conservation and Utilization of Subtropical Agro-Bioresources and Key Laboratory of Innovation and Utilization of Horticultural Crop Resources in South China (Ministry of Agriculture and Rural Affairs), College of Horticulture, South China Agricultural University, Guangzhou, Guangdong 510642, China; 2 Fruit Research Institute, Fujian Academy of Agricultural Sciences, Fuzhou, Fujian 350013, China; 3 Institute of Horticulture, Zhejiang Academy of Agricultural Sciences, Hangzhou, Zhejiang 310021, China

## Abstract

Fruit weight is an integral part of fruit quality and directly influences the commodity value and economic return of fruit crops. Despite the importance of fruit weight, its underlying molecular mechanisms remain understudied, especially for perennial fruit tree crops such as cultivated loquat (*Eriobotrya japonica* Lindl.). Auxin is known to regulate fruit development, but its role and metabolism during the development of loquat fruit remain obscure. In this study, we used a multi-omics approach, integrating whole-genome resequencing-based quantitative trait locus (QTL) mapping with an F_1_ population, population genomics analysis using germplasm accessions, transcriptome analysis, and metabolic profiling to identify genomic regions potentially associated with fruit weight in loquat. We identified three major loci associated with fruit weight, supported by both QTL mapping and comparative genomic analysis between small- and large-fruited loquat cultivars. Comparison between two genotypes with contrasting fruit weight performance by transcriptomic and metabolic profiling revealed an important role for auxin in the regulation of fruit development, especially at the fruit enlargement stage. The multi-omics approach identified homologs of *ETHYLENE INSENSITIVE 4* (*EjEIN4*) and *TORNADO 1* (*EjTRN1*) as promising candidates for the control of fruit weight. Three single nucleotide polymorphism (SNP) markers were also closely associated with fruit weight. Results from this study provide insights into the genetic and metabolic controls of fruit weight in loquat from multiple perspectives. The candidate genomic regions, genes, and sequence variants will facilitate our understanding of the molecular basis of fruit weight and lay a foundation for future breeding and manipulation of fruit weight in loquat.

## Introduction

Fruits are rich in a variety of nutrients needed by humans to maintain a healthy life, including vitamins, trace elements, and sugars [1]. Fruit weight or size is one of the major factors that influence the quality and commodity value of fruits [2]. Fruits with higher weight or larger size often have a higher commodity value and generate greater economic returns. Therefore, revealing the mechanisms of fruit development that underlie fruit-quality traits such as weight/size has been an important research goal in many fruit tree species. Fruit weight or size is influenced by many factors, such as genetics, environment, their interactions, and cultural practices [3]. Plant hormones have been recognized as one of the major factors that affect fruit development [4]. Many studies have shown that auxin is an important hormone influencing fruit size, which is determined by cell number and cell size, through its regulation of cell division and cell expansion [5–7]. A study in apple revealed that higher endogenous auxin levels may increase fruit size, and increased cell size and fruit size were observed after treatment with 1-naphthylacetic acid (NAA) [8]. Similarly, the external application of auxin promoted fruit development and increased fruit diameter in satsuma mandarin and strawberry [9, 10]. Apart from auxin, many other plant hormones also play key roles in fruit growth and development, such as gibberellic acid (GA) [11], cytokinin (CK) [12], ethylene (ETH) [13], abscisic acid (ABA) [14], and brassinosteroids (BRs) [15].

Developmental regulation by endogenous auxin in plants is mediated by many processes, including auxin metabolism, i.e. the biosynthesis and conjugation, transport, and signaling of auxin, the balance of which leads to auxin homeostasis [7, 16]. The biosynthesis of the predominant form of auxin, indole acetic acid (IAA), involves tryptophan aminotransferase of Arabidopsis/tryptophan aminotransferase-related (TAA1/TAR) and flavin monooxygenase (YUCCA) [17]. The conjugation of IAA with amino acids is mediated by proteins like the Gretchen Hagen 3 (GH3) family [18]. PIN and AUX/LAX proteins are essential for the polar transport of auxins, which is required for tissue-specific auxin distribution [19]. Aux/Indole-3-Acetic Acid (Aux/IAA) proteins are repressors of auxin-responsive reporter genes, and their degradation rate is modulated by auxin levels [20]. At low auxin concentrations, the signal transduction is inhibited by Aux/IAAs that repress the transcriptional activities of auxin response factors (ARFs) [21]. At higher auxin concentrations, the proteasomal degradation of Aux/IAA transcriptional repressors is mediated by the transport inhibitor response 1/auxin signaling F-BOX protein (TIR1/AFB) [22]. Subsequently, ARFs are released, activating auxin signaling [23]. Many genes associated with hormone regulation have been identified in tomato, a model species for fruit development and ripening [24]. Silencing of *SlIAA17* in tomato led to larger fruits compared with the wild type, and this was caused by increased cell size and thicker pericarp [25]. In the same study, cell expansion was found to be related to higher ploidy levels, suggesting an interruption of the endoreduplication process. Silencing of *SlPIN4* in tomato led to parthenocarpic fruits, suggesting a role for PIN proteins in the auxin regulation of fruit set [26]. ARFs are members of the auxin signaling pathway that play an important role in fruit development by repressing or activating the transcription of auxin-responsive genes [27, 28]. *SlARF7* negatively regulates fruit set and development by modulating auxin responses [29]. *SlARF9* plays a negative role in cell division at early fruit developmental stages, and increased *SlARF9* expression in transgenic plants led to smaller fruits compared with the wild type [30]. Genes that control the levels of other endogenous hormones also play a role in fruit development. For example, in kiwifruit, the cytokinin biosynthetic gene (*IPT*) increased fruit growth by promoting cell division and expansion [31].

Quantitative trait locus (QTL) mapping has been widely used to identify genomic regions and genes associated with fruit weight and size. The first QTL controlling fruit size, *FRUIT WEIGHT2.2* (*FW2.2*), was identified and cloned in tomato [32]. *fw2.2* encodes a negative regulator of cell division, and changes in its regulation caused differences in fruit size [33, 34]. In addition to *FW2.2*, a series of other loci controlling fruit weight were also identified, such as *FW1.1*, *FW2.1*, *FW3.3*, *FW4.1*, *FW4.2*, and *FW11.2* [35,36]. *SlKLUH*, a gene underlying *FW3.2* in tomato, was found to control fruit and seed weight by regulating cell proliferation in the pericarp [37, 38]. *FW11.3* was reported to control fruit weight by increasing the cell size [39]. However, in perennial crops, QTL mapping is more challenging because of their long juvenile phase and the high costs of maintaining a mapping population. Currently, the QTL mapping approach has been used in several perennial crops, such as apple [40, 41], pear [42, 43], peach [44, 45], grapevine [46], and blueberry [47], to identify loci that underlie fruit weight/size. These studies revealed the complex and polygenic nature of the fruit weight/size trait, which is also influenced by environmental factors.

Transcriptome sequencing is an efficient approach to understanding the transcriptional regulation associated with fruit weight/size. A study in pear compared transcriptome profiles between two accessions with contrasting fruit size at three early fruit developmental stages [48]. Their results showed that the difference in fruit size was mainly caused by a difference in cell numbers and that small fruit size was potentially associated with a short cell division period due to early degradation of cytokinin and gibberellin [48]. Another comparative transcriptome study in pear revealed that auxin signal transduction was important for the fruit enlargement stage [49]. Similarly, in grapevine, transcriptome analysis of individuals with different berry weight performance showed that genes related to auxin metabolism were upregulated in the large-berry genotypes, suggesting an important role for auxin in cell expansion [50].

Current knowledge about the regulatory mechanisms of fruit weight/size is mainly derived from annual plants, whereas fewer studies are available for perennial plants. Cultivated loquat (*Eriobotrya japonica* Lindl.; 2n = 2x = 34) is a subtropical evergreen fruit tree species from the Rosaceae family that is native to China [51, 52]. It has been cultivated for over 2000 years in China for its fleshy and tasty fruit and medicinal properties, such as moisturizing the lungs, relieving coughs, and reducing phlegm [53]. Larger fruits are always desired in the marketplace and have been an important goal for loquat breeding programs. Plant growth regulators, such as the synthetic auxins 2,4-DP [54], naphthalene acetic acid [55], and 3,5,6-TPA [56], have been applied to obtain larger loquat fruits. Understanding the molecular mechanisms that underlie fruit weight/size control will provide guidance for the manipulation of fruit weight/size and facilitate the breeding of large-fruited varieties of loquat. To date, QTL mapping of fruit weight and transcriptome profiling to directly compare small- and large-fruited accessions have rarely been reported in loquat. Previously, our group investigated correlations between cell number/size and fruit weight in 13 loquat accessions and found that cell size may play a predominant role in determining fruit weight [57, 58]. In addition, a repressor of fruit enlargement, *EjBZR1*, was identified, which binds to the *EjCYP90* promoter to repress its expression and cell enlargement [58]. However, the genetic basis and regulatory network that control fruit weight remain unclear. More studies are needed to understand the genetic mechanisms that control fruit weight in loquat.

In this study, we used a combination of QTL mapping (using a bi-parental F_1_ population), population genomic analysis (using germplasm accessions), transcriptome profiling, and metabolic profiling to identify candidate genomic regions and shed light on mechanisms associated with fruit weight in loquat. Results from this study reveal insights into the genetic and metabolic controls of fruit weight in loquat and provide abundant resources for molecular breeding.

## Results

### Phenotypic data of the F_1_ population

The fruit weight phenotype data from an F_1_ population (130 F_1_ plants) derived from crosses between “Ninghaibai” (female parent) and “Dafang” (male parent), two loquat cultivars from China (Zhejiang) and Japan, respectively, were directly retrieved from our previous report [59] (Table S1). In brief, the average fruit weight (g) of 30 mature fruits from each plant was used for analysis. The two parental plants had similar fruit weights (Table S1), but extensive variation was observed in the F_1_ population (Fig. S1A, B). As previously reported, fruit weight showed a near-normal distribution (Fig. S1B, C), indicating the quantitative nature and polygenic genetic control of this trait. The phenotype data were recorded for only one year in 2020, owing to tree losses caused by cold damage in 2021.

### Genotyping and high-density genetic map construction

Through whole-genome resequencing (WGRS), more than 21 Gb of sequencing data (>27.00× coverage) were obtained for each parent, and an average of 8 Gb of sequencing data per sample (9.57× coverage) were obtained for the F_1_ individuals (Table S1). The reference genome of “Seventh Star” [60] was used for analysis, as it was first released to the public at the initiation of this project. However, both the “Seventh Star” and “Jiefangzhong” [52] (released in 2021) genomes were used for the downstream candidate gene search. The average overall alignment rate was 97.17%. Variant calling and further filtering yielded a total of 2 184 538 single nucleotide polymorphisms (SNPs) between the two parents, with a Ti/Tv ratio of 2.48.

**Figure 1:**
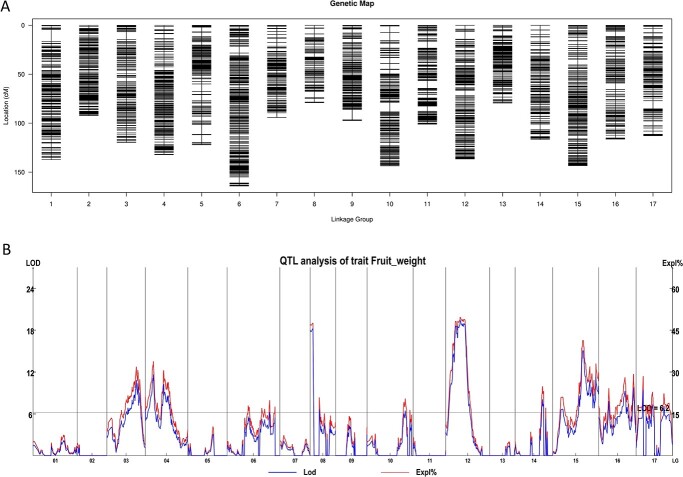
Distribution of markers in the high-density genetic map and quantitative trait loci associated with fruit weight. (A) The X-axis corresponds to each linkage group; the Y-axis represents the genetic distance (cM). (B) The X-axis represents each linkage group; the blue line corresponds to the LOD score on the left Y-axis; the red line corresponds to the phenotypic variation explained (%) on the right Y-axis. The horizontal grey line indicates the threshold of LOD with 99.5% confidence interval (LOD = 6.2).

The identified SNPs were used for genetic map construction with a Bin map method and HighMap software [61]. The SNPs were assigned to groups based on their physical locations, and SNPs within each group were partitioned into bins after performing pair-wise linkage analysis and corrections of genotypes based on linkage phase. SNPs within the same bin were considered to exhibit no recombination. A consensus map was constructed using fully informative markers/bins with phasing information available for both alleles for each parent. Finally, a high-density genetic map composed of 17 linkage groups (LGs) and 3859 bins and spanning a genetic distance of 1988.12 cM was obtained (Fig. 1A). The length of each LG ranged from 79.05 cM to 164.16 cM, with an average density of 0.52 cM/marker (Table 1). According to collinearity analysis, the Spearman correlation coefficients of all LGs were ≥ 0.9980 (Fig. S2), indicating high consistency between the reference genome and the LGs, as well as high accuracy of the recombination rate calculations.

**Table 1:** Summary statistics for 17 linkage groups

Linkage group	No. of loci (bins)	Map distance (cM)	Average distance between loci (cM)	Maximum gap (cM)	Gaps <5 cM (%)
1	210	137.25	0.65	12.13	99.52
2	241	92.32	0.38	2.36	100.00
3	191	119.65	0.63	7.90	99.47
4	343	132.16	0.39	4.84	100.00
5	203	122.06	0.60	10.20	98.02
6	349	164.16	0.47	7.01	99.43
7	164	94.24	0.57	4.84	100.00
8	127	79.05	0.62	6.13	98.41
9	209	97.35	0.47	10.68	99.52
10	282	143.59	0.51	9.73	98.22
11	165	101.00	0.61	7.90	98.17
12	292	136.56	0.47	7.90	99.31
13	207	79.46	0.38	4.84	100.00
14	179	116.79	0.65	5.27	99.44
15	288	143.41	0.50	5.70	99.65
16	238	116.24	0.49	6.57	99.16
17	171	112.83	0.66	8.35	99.41
Total	3859	1988.12	-	-	-

### QTL identification for fruit weight

QTLs were identified for fruit weight based on the high-density linkage map and the phenotypic data. In total, 17 QTLs were detected on eight LGs, corresponding to eight chromosomes based on the “Seventh Star” genome, including Chr3, Chr4, Chr8, Chr12, Chr14, Chr15, Chr16, and Chr17 (Fig. 1B, Table 2). The large number of QTLs reflected the complex and polygenic nature of fruit weight. Among these QTLs, we identified three major QTLs on Chr8, Chr12, and Chr15, which had a phenotypic variation explained (PVE) value ranging between 20.0% and 49.7%. Sequences surrounding the flanking markers of the QTLs were extracted from the “Seventh Star” genome and compared to the “Jiefangzhong” genome to identify the corresponding coordinates. Both the physical sizes and the numbers of annotated genes of the corresponding genomic regions of these 17 QTLs were highly similar between the two reference genomes (Table S2). The physical sizes of the 17 QTLs ranged from 0.09 to 23.16 Mb and covered a total of 6113 genes in the “Seventh Star” genome, whereas the physical sizes ranged from 0.09 to 23.29 Mb and covered 6249 genes in the “Jiefangzhong” genome.

**Table 2:** Summary information for quantitative trait loci associated with fruit weight

Linkage group	Start (cM)	End (cM)	No. of bins	LOD range	PVE range (%)
					
3	67.518	105.182	79	6.22–10.86	19.8–31.9	
3	65.587	66.36	6	6.41–6.87	20.3–21.6	
3	60.544	61.319	2	6.21–6.38	19.7–20.2	
4	8.272	29.533	20	6.38–11.66	20.2–33.8	
4	51.836	76.221	143	6.68–10.29	21.1–30.6	
8	0	28.415	6	6.63–18.25	20.9–47.6	
12	14.231	74.782	124	6.31–19.4	20.0–49.7	
14	83.081	88.899	12	6.94–8.1	21.8–24.9	
15	80.128	143.41	164	6.3–15.07	20.0–41.4	
15	25.859	32.088	7	6.61–6.71	20.9–21.1	
16	31.388	32.163	5	6.81–7.83	21.4–24.2	
16	72.154	83.911	9	6.71–9.29	21.1–28.1	
16	87.021	90.502	14	6.35–6.98	20.1–21.9	
16	106.093	110.748	11	7.41–9.78	23.1–29.3	
17	86.08	87.624	7	6.53–7.11	20.7–22.3	
17	21.725	22.111	2	9.46–9.48	28.5–28.5	
17	47.753	52.401	14	6.23–7.33	19.8–22.9	

### Population genomics of small- and large-fruited cultivars

**Figure 2:**
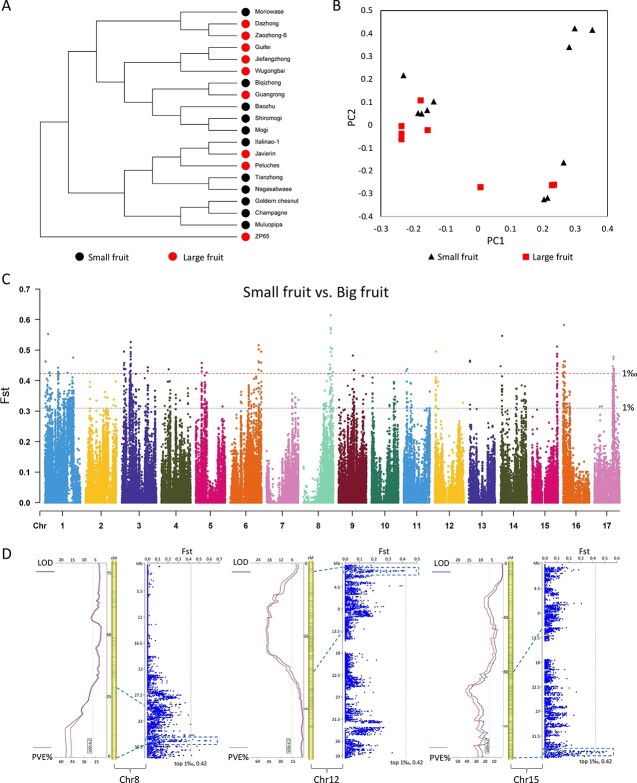
Genetic relationship and population differentiation of small- and large-fruited cultivars, and comparison of QTL regions and top *F*_st_ regions potentially associated with fruit weight. (A) and (B) The black color indicates small-fruited cultivars; the red color indicates large-fruited cultivars. The phylogenetic tree was constructed using PHYLIP with 100 bootstraps and visualized using MEGAX software. Principal component analysis (PCA) was carried out using PLINK. (C) Distribution of *F*_st_ comparing small- and large-fruited cultivars. Each dot represents a sliding window of 10 kb, within which the average *F*_st_ value was calculated. The blue line is the top 1% *F*_st_ line (*F*_st_ = 0.31); the brown line is the top 0.1% *F*_st_ line (*F*_st_ = 0.42). (D) Comparisons for Chr8, Chr12, and Chr15, which contain three major QTLs for fruit weight.

To gain additional evidence for identification of the genomic regions potentially associated with fruit weight, we also obtained WGRS data for a total of 20 commonly known small- or large-fruited loquat cultivars (Table S3). WGRS data were generated in this study for 15 genomes, with an average coverage of 10.85×, and we also utilized published WGRS data for five genomes with an average coverage of 8.45×. Variant calling and filtering identified a total of 3 697 866 SNPs, which were used for the following analyses. Phylogenetic analysis revealed that small- or large-fruited cultivars were not specific to a single branch or a cluster (Fig. 2A, B). Although these germplasm accessions were limited in number, they represented diverse genetic backgrounds or clusters. Calculation of *F*_st_ using a sliding window approach identified genomic regions that were highly genetically differentiated between the small- and large-fruited groups. By focusing on genomic regions above the top 1% *F*_st_ line (*F*_st_ = 0.31), we found that a large proportion of Chr1 was highly differentiated between the two groups. Moreover, we noticed a region of Chr8 that contained the highest peak above the top 0.1% *F*_st_ line (*F*_st_ = 0.42). Interestingly and importantly, this highly differentiated region between the two groups on Chr8 overlapped with a major QTL associated with fruit weight on the same chromosome (Fig. 2D). Similarly, the highest *F*_st_ peaks on Chr12 and Chr15 also overlapped with two major QTLs. In summary, the overlapped regions on Chr8, Chr12, and Chr15, with evidence from both the bi-parental QTL mapping approach and the *F*_st_ analysis of germplasm accessions, may play an essential role in determining the fruit weight of loquat, and they can be prioritized for candidate gene search.

### Comparative transcriptome analysis of two sister lines with contrasting fruit weights

**Figure 3:**
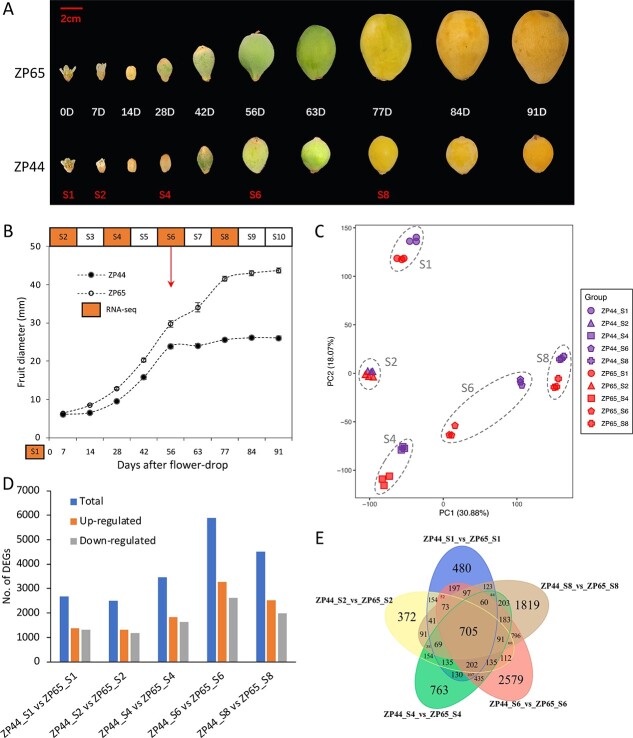
Observation of fruit development and DEGs identified from the RNA-seq experiment. (A) Observations of fruit development of ZP44 and ZP65 at 10 time points, including 0 days past anthesis (0D, S1), 7D (S2), 14D, 28D (S4), 42D, 56D (S6), 63D, 77D (S8), 84D, and 91D. (B) Comparison of fruit diameter (transverse diameter) between ZP44 and ZP65. The values are the mean fruit diameter of 15 fruits. Bars are standard errors. (C) Principal component analysis using transcriptome profiles of all 30 replicates. (D) Summary of DEGs between ZP44 and ZP65 at each stage. (E) Comparison of DEGs at each stage.

To investigate transcriptional regulation related to fruit development and facilitate candidate gene searching, we obtained transcriptome profiles at five different developmental stages for the two sister lines ZP44 and ZP65 (Fig. 3A) derived from a cross between Zaozhong No. 6 (female parent) and Peluches (male parent). ZP44 had extremely small fruits (11–15 g), whereas ZP65 had extremely large fruits (up to 82.69 g). These two genotypes had a relatively larger fruit weight difference than those observed in the F_1_ population. Their fruit developmental processes were observed by measuring the fruit diameter (transverse diameter) owing to its high correlation coefficient (0.964) with fruit weight. Data were recorded at 0, 7, 14, 28, 42, 56, 63, 77, 84, and 91 days past anthesis (DPA) (Fig. 3B). The fruit size difference between the two genotypes was small at the earlier stages. However, the fruit diameter of ZP44 reached a plateau at 56 DPA, whereas fruits of ZP65 continued to enlarge and reached a plateau at 77 DPA. Five representative stages were selected for transcriptome sequencing: 0 (S1), 7 (S2), 28 (S4), 56 (S6), and 77 (S8) DPA. Phenotypic observations revealed that S6 was a key developmental stage at which the fruit development of ZP44 and ZP65 diverged and became visibly different.

An average of 7 Gb of transcriptome sequencing data per sample were obtained for a total of 30 samples (2 genotypes × 5 stages × 3 biological replicates) (Table S4). To utilize and improve the gene annotation of the “Jiefangzhong” reference genome generated by our group, reads were mapped to the “Jiefangzhong” genome with an average overall mapping rate of 95.01%. Consistent with the phenotypic observations, the largest difference between ZP44 and ZP65 was observed at stage S6, according to the principal component analysis of the 30 transcriptome profiles (Fig. 3C). The differentially expressed genes (DEGs) between ZP44 and ZP65 at each stage were identified and compared (Table S5). Similarly, S6 had the largest number of DEGs, with 3267 upregulated genes and 2623 downregulated genes (Fig. 3D), and it also had the largest number of stage-specific DEGs (2579 for ZP44_S6 vs ZP65_S6) (Fig. 3E). Therefore, both phenotypic observations and transcriptome profiling suggested that some unique changes happened at the S6 stage, which may be associated with the large fruit weight difference between the two genotypes. Gene ontology (GO) enrichment analysis was performed for the DEGs between ZP44 and ZP65 at each stage. Strikingly, eight hormone-related GO terms were significantly enriched for the DEGs at S6, including “regulation of hormone levels”, “hormone metabolic process”, “cellular response to auxin stimulus”, “auxin-activated signaling pathway”, “response to gibberellin”, “hormone catabolic process”, “response to auxin”, and “hormone biosynthetic process” (Table S6). Interestingly, the three auxin-related GO terms were only enriched specifically at the S6 stage, suggesting that auxin-related genes may play an important role at this key stage when the fruit is enlarging. In addition to auxin and gibberellin, we also noticed the enrichment of salicylic acid-related GO terms at the S4 stage, indicating the role of this hormone at the early fruit developmental stage. The pathway enrichment analysis identified numerous sugar-related pathways significantly enriched at the S6 stage (Table S7), suggesting a difference in sugar accumulation corresponding to the difference in fruit size between the two genotypes.

**Figure 4:**
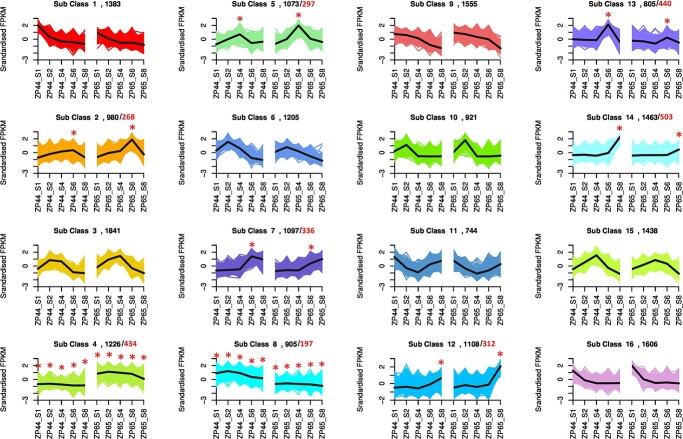
K-means clustering of DEGs. The number in black font is the number of genes in each sub-class; the number in red font is the number of DEGs between ZP44 and ZP65 for the “star” labeled time point.

To further identify the different expression patterns between ZP44 and ZP65 during fruit development, the DEGs were clustered into groups using the K-means method (Table S8, Fig. 4). A total of 16 sub-classes or types were identified, among which eight sub-classes showed clearly different expression patterns between the two genotypes; these included sub-classes 2, 4, 5, 7, 8, 12, 13, and 14. Sub-classes 4 and 8 contained 434 and 197 genes that were differentially expressed at all stages between the two genotypes. Genes from sub-classes 2, 7, and 13 showed different expression patterns between the two genotypes at the S6 stage. By comparing the genomic locations of the DEGs with the QTLs and top *F*_st_ regions, we found 311 DEGs located only in QTLs, 16 DEGs located only in top 1% *F*_st_ regions, and eight DEGs located in the overlapping regions. These genes with different expression patterns will facilitate subsequent candidate gene searches.

### Auxin metabolite profiling

**Figure 5:**
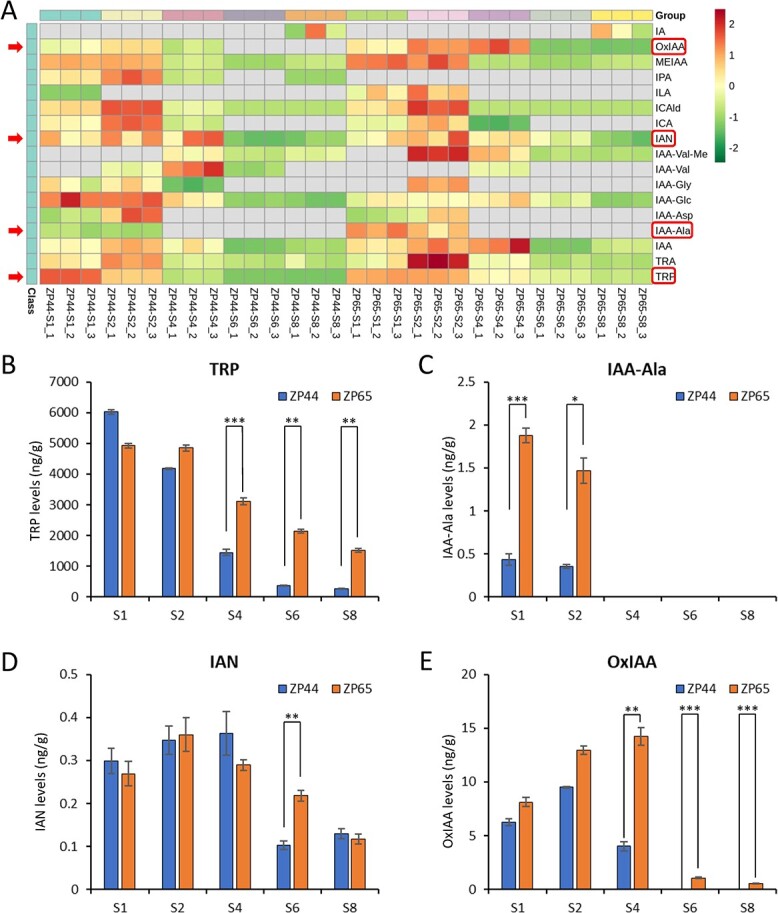
The clustering of auxin metabolites and significantly different metabolites between ZP44 and ZP65. (A) A heat map showing auxin metabolite levels. Data were processed using the z-score normalization method. (B)-(E) Comparisons of metabolite levels for L-tryptophan (TRP), 3-indoleacetonitrile (IAN), N-(3-indolylacetyl)-L-alanine (IAA-Ala), and 2-oxindole-3-acetic acid (OxIAA).

Considering the possibly important role of auxin metabolism in fruit development, auxin metabolic status was investigated in ZP44 and ZP65 at the same five stages (Table S9). A total of 17 types of auxin metabolites were detected in these samples. Clustering based on metabolite levels revealed a general trend in which the levels of most detected metabolites were higher in the first three stages than in the latter two stages (Fig. 5A). The levels of many metabolites decreased significantly when comparing S1 vs S6 or S1 vs S8 within each genotype (Table S10, S11). This result suggested that auxin signaling and metabolism may be more active at earlier fruit developmental stages than at the mature fruit stage. Interestingly, we found significant differences in levels of several metabolites between the two genotypes, including L-tryptophan (TRP), 3-indoleacetonitrile (IAN), N-(3-indolylacetyl)-L-alanine (IAA-Ala), and 2-oxindole-3-acetic acid (OxIAA) (Fig. 5B-E). Importantly, the precursor of auxin, TRP, was still present at high levels at stages S4, S6, and S8 in ZP65, indicating high auxin levels, whereas TRP levels decreased sharply in ZP44. Therefore, consistent with findings from the transcriptome analysis, auxin metabolite profiling validated the presence of higher auxin levels in ZP65 than in ZP44, especially at the fruit enlargement stage. These results suggested that auxin metabolism is one of the key factors that determine the difference in fruit weight/size between ZP44 and ZP65.

### Candidate genes and markers potentially associated with fruit weight

**Figure 6:**
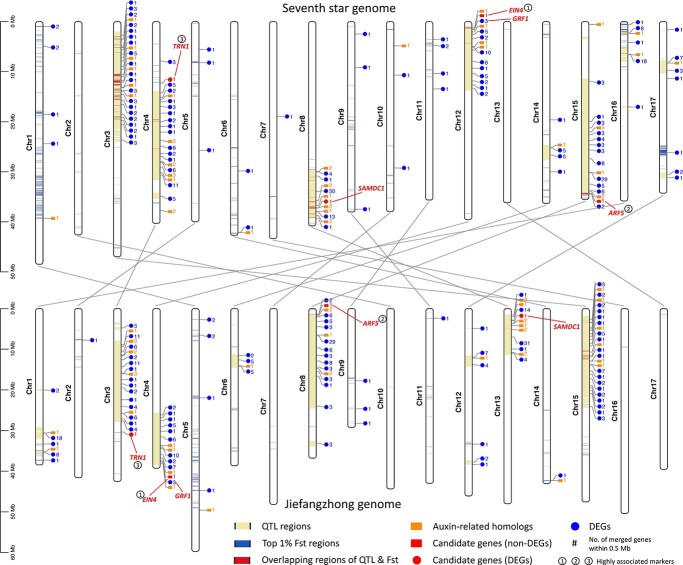
Distribution of QTL regions, top 1% *F*_st_ regions, and candidate genes potentially associated with fruit weight in two reference genomes of loquat. The explanations for color codes of regions and genes are at the bottom of the figure. The connecting lines between the two genomes indicate the same chromosomes.

To search for candidate genes potentially associated with fruit weight, the following criteria were considered: 1) location in QTLs; 2) location within or adjacent to top 1% *F*_st_ regions; 3) differential expression between ZP44 and ZP65; 4) homology to known genes associated with fruit weight or related functions (cell proliferation, organ size, etc.); 5) presence of or proximity to markers highly associated with fruit weight (first assessed based on predicted genotypes from GATK, then validated using Sanger sequencing); and 6) presence of polymorphisms in functional domains between small- and large-fruited genotypes. Because of the potentially different genetic backgrounds of the plant materials used for QTL analysis, *F*_st_ analysis, and RNA-seq, a candidate gene may not necessarily meet all the criteria. After mining, we identified two candidate genes with relatively more evidence, a homolog of *ETHYLENE INSENSITIVE 4* (*EjEIN4*) and a homolog of *TORNADO 1* (*EjTRN1*) (Table 3). Three other genes were also summarized, although with relatively less evidence, including three homologs of *Auxin Response Factor 5* (*EjARF5*), *S-adenosylmethionine decarboxylase proenzyme 1* (*EjSAMDC1*), and *Growth-Regulating Factor 1* (*EjGRF1*) (Table 3). For visualization, the QTLs, top 1% *F*_st_ regions, DEGs located in these regions, candidate genes, and markers highly associated with fruit weight were plotted on a map for both reference genomes (Fig. 6, Table S12, S13).

**Table 3:** Summary of evidence for candidate genes potentially associated with fruit weight in loquat

Evidence	*EjEIN4*	*EjTRN1*	*EjARF5*	*EjGRF1*	*EjSAMDC1*
Within QTLs	Yes	No	Yes	Yes	Yes
Within or adjacent to top 1% *F*_st_ regions	Yes	Yes	Yes	Yes	Yes
Differentially expressed between ZP44 & ZP65	No	Yes	No	Yes	Yes
Homologous to known genes associated with fruit weight or related functions	Yes	Yes	Yes	Yes	Yes
Contain markers highly associated with fruit weight	Yes	Yes	Yes	Close^a^	NA
Contain polymorphisms in functional domains between small- and large-fruited genotypes	Yes	Yes	No	NA	NA

a Marker 1 is 127 731 bp away from *EjGRF1*.

**Figure 7:**
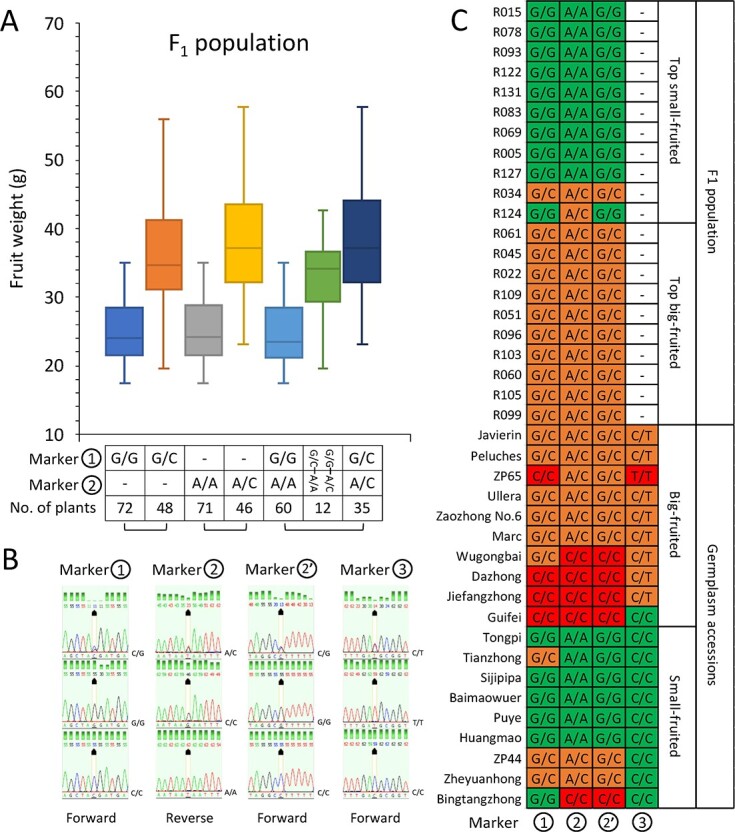
Single nucleotide polymorphism markers closely associated with fruit weight. (A) The distributions of fruit weight for F_1_ individuals with predicted genotypes from GATK for Marker 1 and 2. The predicted genotypes of Marker 2 were used for the comparison because there were more available data for the minor class of genotype combinations than for Marker 2′. The genotypes of Dafang were “C/G” at Marker 1 and “A/C” at Marker 2. The genotypes of Ninghaibai were “G/G” at Marker 1 and “A/A” at Marker 2. The number of plants in each column corresponds to the genotype combination in that column. (B) Three genotypes at the four SNP loci based on Sanger sequencing from the validation panel. (C) The genotypes obtained from Sanger sequencing for the validation panel at Markers 1, 2, 2′, and 3.

In total, three SNP markers highly associated with fruit weight, Markers 1, 2 (2′), and 3, were identified using a validation panel composed of the extremely small- and large-fruited F_1_ individuals, as well as commonly known small- and large-fruited loquat cultivars/accessions (Fig. 7A-C). Marker 1 was identified 395 bp upstream of *EjEIN4*. Markers 2 and 2′ were located 1524 bp upstream of *EjARF5* and within its 8^th^ intron, respectively. Marker 3 was located in the coding region of *EjTRN1*. Alleles of Marker 1 and 2 (2′) segregated in the F_1_ population, whereas the genotype of Marker 3 was fixed for the F_1_ population and only showed high association with fruit weight in the natural germplasm accessions. Using the predicted genotypes of Marker 1 and 2 from GATK, the fruit weights for F_1_ individuals with different genotype combinations were compared. Marker 2 was used for the comparison because there were more data available for the minor class of genotype combinations than for Marker 2′. The results showed that individuals with “G/G and A/A” had lower fruit weight, those with “G/C and A/C” had higher fruit weight, and those with only one “large-fruited” genotype (either “G/C” and “A/A” or “G/G” and “A/C”) were somewhere in the middle (Fig. 7A). These results demonstrated high correlations between fruit weight and the identified markers.

**Figure 8:**
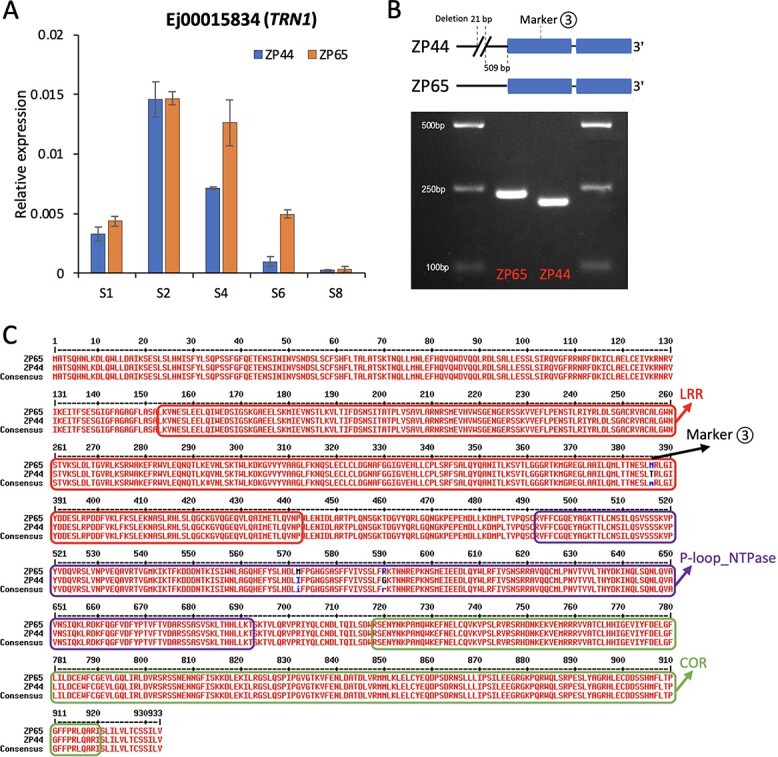
The differential expression patterns and polymorphisms of *EjTRN1* between ZP44 and ZP65. (A) Relative expression levels of *EjTRN1* at five developmental stages of ZP44 and ZP65. (B) An insertion/deletion identified at the promoter of *EjTRN1* with PCR validation. (C) Comparison of protein sequences of *EjTRN1* between ZP44 and ZP65. The functional domains were predicted using the conserved domain search tool at NCBI.

To investigate the polymorphisms in the gene regions, we obtained the full-length sequences for the three genes tagged with markers highly associated with fruit weight (*EjEIN4*, *EjTRN1*, and *EjARF5*). The whole gene region of *EjTRN1* was deeply covered by uniquely mapped Illumina reads for ZP44 and ZP65 (both are homozygous for the whole gene region). Therefore, the sequences were manually retrieved by visualizing the alignment file. For *EjEIN4* and *EjARF5*, we aligned the 3^rd^ generation PacBio HiFi reads of Puye (available in-house from our genome projects) to the Jiefangzhong reference genome and manually retrieved the gene sequences by visualizing the alignments. The accession Puye was used because of its homozygous “small-fruited” genotypes at Markers 1 and 2, as well as the availability of HiFi read data. Strikingly, *EjTRN1* turned out to be a highly promising candidate gene. In Arabidopsis, *TRN1*, an auxin-signaling pathway gene, is involved in auxin transport, cell proliferation, and plant growth [62]. *EjTRN1* was differentially expressed between ZP44 and ZP65 (Fig. 8A). Consistent with this result, we identified a large 21-bp deletion in the *EjTRN1* promoter in ZP44, which was also validated using PCR and gel electrophoresis (Fig. 8B). By comparing the predicted protein sequences of ZP44 and ZP65, we found that Marker 3, a SNP marker, led to an amino acid change (M/T) within the leucine-rich repeat (LRR) domain, making it a functional marker, and this amino acid change was also highly associated with fruit weight (Fig. 7C). After genotyping the validation panel at the Indel locus, we found that only ZP44 and Tongpi, two small-fruited accessions, contained the homozygous “deletion” genotype, although a few other accessions also carried the “deletion” (Fig. S3). Consequently, it turned out to be Marker 3 that was highly correlated with fruit weight rather than the Indel locus, although it is also likely that this Indel may contribute to the small-fruited phenotype by influencing the expression of *EjTRN1*. In Arabidopsis, *EIN4*, an ethylene receptor gene, was reported to interact with *SAUR76*, *SAUR77*, and *SAUR78*, which are associated with plant growth and cell expansion [63]. Interestingly, by comparing the predicted protein sequence of *EjEIN4* between Jiefangzhong and Puye, two homozygous “large-fruited” and “small-fruited” genotypes at Marker 1, we identified at least one amino acid difference within each of its domains (Fig. S4). Based on these results, *EjTRN1* and *EjEIN4* were considered to be two promising candidates associated with fruit weight in loquat.

In tomato, *SlARF5* was reported to regulate fruit development and influence fruit size and weight [64]. For *EjARF5*, we obtained the sequences of two haplotype alleles of Puye that differed only at one amino acid towards the end of the predicted protein (Fig. S5). By comparing these protein sequences from Puye (the small-fruited genotype) with that from Jiefangzhong (the large-fruited genotype), we identified three amino acid differences and one Indel (two amino acid insertion/deletion) within its coding region (Fig. S5). However, these polymorphisms were not located within its predicted functional domains. It is possible that the causal polymorphism(s) may be at other sites. However, is also possible that another unknown gene close to Marker 2 (2′), rather than *EjARF5*, underlies the fruit weight variation associated with this locus. Future experiments can be designed to further validate its function.

In addition to the above genes, *EjSAMDC1* and *EjGRF1* were differentially expressed between ZP44 and ZP65 (Fig. S6A, B). In apple, *MdSAMDC1* was reported to participate in fruit development and cell growth [65]. Strikingly, the expression of *SAMDC1* is known to be downregulated by auxin in Arabidopsis [66]. By investigating the expression patterns, we found that the expression of the *EjSAMDC1* homolog was significantly much higher in ZP44 than in ZP65 at the S6 stage (Fig. S3). It is likely that auxin levels were much higher in ZP65 at the S6 stage, repressing the expression of *EjSAMDC1*, in contrast to the low auxin levels at the S6 stage in ZP44, in which the expression of *EjSAMDC1* was not repressed. *EjGRF1* was also adjacent to Marker 1 (127 731 bp away), which was highly associated with fruit weight. In Arabidopsis, *GRF1* was reported to play a role in the regulation of cell expansion and to influence organ sizes [67, 68]. Therefore, these two genes may also be worthy of future in-depth investigation. Collectively, the multi-omics approaches identified several candidate genes that are potentially associated with fruit weight in loquat.

## Discussion

Fruit weight is an integral part of fruit quality for fruit tree crops such as loquat and directly influences commercial value and consumer acceptance. Despite the importance of fruit weight, its associated markers and underlying genetic mechanisms, which could greatly facilitate the breeding of cultivars with optimal fruit weight performance, remain understudied in loquat. This is largely due to a major obstacle specific to perennial trees: they usually must undergo a juvenile phase of many years before entering the reproductive stage and bearing fruit. Plant hormones, especially auxin, have been reported to play important roles in the regulation of fruit development [69, 70]. However, the role of auxin and its metabolism remain largely unexplored in loquat. To address these questions, we used a multi-omics approach, integrating WGRS, transcriptome sequencing, and metabolite profiling to identify genomic regions potentially associated with fruit weight. Our results revealed insights into the genetic and metabolic controls of fruit weight and provided abundant resources of genomic regions, genes, and sequence variants that will enable future in-depth investigation and facilitate the breeding of loquat.

High-density genetic maps are an important genomic resource and a prerequisite for QTL mapping and map-based cloning [40, 45, 71, 72]. However, few genetic mapping studies have been available in loquat. With WGRS data (~10× coverage), we were able to genotype an F_1_ population in a genome-wide manner. Because of the large number of co-segregating SNPs, we used a Bin method that has been used previously [61, 73, 74] to group SNPs without recombination into the same bin, and we created a consensus map. Previously, a high-density genetic map was constructed for bronze loquat; it was composed of 960 loci and spanned 1707.4 cM with a density of 1.78 cM/marker [72]. The genetic map in the present study spanned 1988.12 cM and covered all 17 chromosomes with a density of 0.52 cM/marker, considerably higher than that in previous studies based on amplified fragment length polymorphisms (AFLPs), simple sequence repeats (SSRs), or SNPs using RAD-seq [72, 75, 76].

The high-density genetic map and phenotypic data enabled the identification of 17 QTLs potentially associated with fruit weight in loquat. The number of QTLs for loquat fruit weight seems to be higher than that for several other crops, such as blueberry (seven QTLs) [47], grapevine (six QTLs) [46], peach (four QTLs) [45], and apple (two QTLs) [42]. There are several possible reasons for this larger number. First, it may reflect the complex and polygenic nature of the trait or the nature of the crop. The different genetic backgrounds of parental genotypes used in these studies may also explain the QTL number differences, as fewer loci may be identified if the parental genotypes are fixed at some loci that do not segregate in the population. Second, it may be attributable to the limited number of F_1_ individuals, which may only reveal a rough genomic region, or to the limitation of a single year of phenotypic data. However, we did observe three major QTLs that explained 20.0%–49.7% of the phenotypic variation. Compared with QTLs with minor effects [45, 47], major effect QTLs appear to be more stable across different years or environments, as demonstrated in grapevine [46]. The major fruit weight QTL with 61% PVE in that study was stable across different studies [77, 78]. The QTLs identified in the current study can be further validated in other populations or compared with other studies in the future to identify stable QTLs.

Considering the large number of QTLs and the relatively large corresponding genomic regions, we also used WGRS data from commonly known small- or large-fruited loquat cultivars or accessions from the natural population preserved in the germplasm repository, whose fruit weight has been tested for many years. Corroborating our QTL results, the top *F*_st_ peak on Chr8 was highly consistent with the QTL peak on the same chromosome. Therefore, the *F*_st_ analysis based on loquat germplasm accessions with diverse genetic backgrounds supports the existence of a major locus associated with fruit weight on the lower part of Chr8. It is highly possible that the alleles controlling fruit weight at this locus are prevalent in loquat germplasm. Similarly, the *F*_st_ peaks on Chr12 and Chr15 and located within the two major QTLs also support the existence of two other major loci associated with fruit weight. We believe that the three loci on Chr8, Chr12, and Chr15 play an important role in controlling fruit weight in loquat, as evidenced by both QTL-mapping and *F*_st_ analysis. Apart from these three major loci, we also observed many genomic regions that were highly differentiated between small- and large-fruited loquat accessions on other chromosomes, consistent with the large number of QTLs identified by the bi-parental QTL mapping approach. Collectively, the overlapping regions between QTLs and top *F*_st_ regions are good candidate genomic regions that potentially control fruit weight in loquat.

However, the results from the QTL analysis, *F*_st_ analysis, and RNA-seq analysis should be interpreted with caution, as different materials were used in these analyses, although the two materials used for RNA-seq (ZP44 and ZP65) are offspring of two large-fruited accessions (Zaozhong No. 6 and Peluches) included in the *F*_st_ analysis. We propose that the overlapping results between the QTL analysis and *F*_st_ analysis could be a coincidence but may also have significant relevance and implications. The germplasm accessions used for *F*_st_ analysis represent diverse genetic backgrounds, probably covering most of the gene pool for fruit weight/size. By comparison, the two parental cultivars used for the QTL analysis represent relatively narrower genetic backgrounds, and it is likely that some genes/alleles controlling fruit weight/size may already be fixed (not segregating) in the F_1_ population. From this perspective, the overlapping results could be a coincidence, as it is unknown which genes from the whole gene pool are represented or segregating in the F_1_ population. An example would be Marker 3 (located within *EjTRN1*), whose genotypes/alleles are fixed or not segregating in the F_1_ population but are highly associated with fruit weight in the germplasm validation panel. From another perspective, the genomic regions with high *F*_st_ values are highly differentiated between the small- and large-fruited groups. These *F*_st_ peaks were captured because the SNP alleles in these regions are quite different between the two groups. Therefore, the overlapping results could cover genes/alleles that were not only segregating in the F_1_ population but also common/universal in the loquat germplasm. In other words, this could cover genes/alleles that were recombined after human selection and breeding, during which the large-fruited alleles may have accumulated in the large-fruited group, whereas the small-fruited alleles may have accumulated in the small-fruited group. Both Marker 1 and Marker 2 (located in both QTLs and top *F*_st_ regions) can serve as examples, as they are highly associated with fruit weight for both the F_1_ individuals and the germplasm accessions in the validation panel.

Because the two materials used for RNA-seq, ZP44 and ZP65, differ from those in the QTL and *F*_st_ analyses, the genes underlying fruit weight/size in the F_1_ population or in the germplasm accessions may not necessarily be differentially expressed between ZP44 and ZP65. Therefore, when searching for candidate genes, we not only focused on DEGs but also used a series of other criteria, such as whether they were tagged with markers highly associated with fruit weight or homologous to known genes associated with fruit weight. Given that ZP44 and ZP65 were derived from a cross between two large-fruited cultivars, it is highly likely that they are of the “large-fruited” genotype at many loci, including those tagged with Marker 1 and Marker 2. Supported by abundant evidence, *EjTRN1* (tagged with Marker 3) may be one of the promising candidate genes that can explain the extremely small fruit weight of ZP44.

An important role of auxin in regulating fruit weight has been revealed from both transcriptomic and metabolic perspectives. To facilitate the candidate gene search, we performed comparative transcriptome analysis of two sister lines, ZP44 and ZP65, that were derived from a cross between two commonly known large-fruited cultivars, Zaozhong No. 6 and Peluches. It is interesting that the cross between two large-fruited cultivars led to ZP44, an extremely small-fruited line. Therefore, genes or alleles from Zaozhong No. 6 and Peluches may have recombined in ZP44, which probably accumulated alleles leading to small fruits. Both phenotypic observations and transcriptome profiling of these two contrasting lines suggested that the key divergence point leading to fruit size differences occurred at the S6 stage when the fruits were quickly expanding. Interestingly, both functional enrichment analysis and metabolomics analysis revealed a key role for auxin at this important stage. Auxin is an important plant hormone that is closely involved in the initiation of fruit development and determines fruit size by regulating cell division and expansion [7, 29]. Its vital role in fruit development is implied by the fact that the highest auxin levels are usually observed in developing fruits compared with other parts of the plant [79]. Consistent with this notion, we also observed higher levels of auxin metabolites at earlier fruit developmental stages compared with mature fruit stages. Moreover, a few studies have already reported the effect of auxin application on fruit development in loquat [56, 80]. One study reported that the application of the synthetic auxin 2,4-DP increased the fruit size of loquat and also led to earlier maturation and harvest time [80]. Similarly, another study revealed that treatment with the synthetic auxin 3,5,6-TPA led to larger fruit size by decreasing cell turgor pressure [56]. In the current study, we identified several auxin-related candidate genes, such as *EjTRN1*, *EjARF5*, and *EjSAMDC1* (Table S13). This result implies that variations in auxin-related genes may be recombined through breeding and selection, leading to cultivars with various fruit weights and sizes.

The role of auxin in fruit development is achieved through the regulation of auxin-responsive genes by gene families such as *ARFs*, *Aux/IAAs*, *GH3*, and *Small Auxin Up RNAs* (*SAURs*) [81–84]. Auxin transport also plays a role. In Arabidopsis, different auxin transport patterns were observed in *trn1* and *trn2* mutants compared with the wild type and could lead to twisted growth by changing auxin distributions [62, 85]. In the current study, the expression of *EjTRN1* seemed to decrease to a lower level significantly earlier in ZP44 than in ZP65 during fruit development, implying that auxin transport and distribution may be affected in ZP44 compared with ZP65. This may contribute to a shorter duration of auxin supply in ZP44, as supported by the expression patterns of *EjSAMDC1* as well as the results from metabolomics. In addition to auxin, ethylene may also play an important role in loquat fruit development, as the candidate gene *EjEIN4* is an ethylene receptor gene. The ethylene and auxin signaling genes may act together to regulate fruit development. Therefore, the metabolic status of ethylene may be worthy of future research.

## Conclusion

In this study, combining bi-parental QTL mapping and population genomics analysis of small- and large-fruited accessions, we identified three major loci on Chr8, Chr12, and Chr15 potentially associated with fruit weight, as well as other genomic regions. We developed three SNP markers associated with fruit weight, which can be further applied to the breeding of loquat cultivars with higher fruit weight. Our results revealed that the auxin signaling pathway may have been under selection over the breeding history of loquat, leading to cultivars with various fruit weights. Results from this study provide insights into fruit weight using a multi-omics approach, with perspectives from genomics, transcriptomics, and metabolomics. Overall, the genomic regions, candidate genes, and other results provide abundant and critical information for understanding the genetic basis of fruit weight in this fruit tree crop, and they lay a solid foundation for future breeding and manipulation of fruit weight/size in loquat.

## Materials and methods

### Plant materials and phenotyping

An F_1_ population was obtained from crosses between two loquat cultivars, Ninghaibai (female parent) and Dafang (male parent), which were planted at the Yangdu Scientific Research and Innovation Base of Zhejiang Academy of Agricultural Sciences (30.4383°N, 120.4164°E) in 2012. A total of 130 F_1_ plants that stably formed flowers and bore fruit since 2017 were selected for investigation of fruit weight at the mature fruit stage in May 2020. The plants were grown under natural conditions with regular management, without flower or fruit thinning or bagging. From each tree, 30 loquat fruits were randomly collected for measurement of fruit weight. The details of the phenotyping were described in our previous report [59]. A total of 14 commonly known small-fruited and large-fruited cultivars with publicly available fruit weight information (Table S3) and two sister lines (ZP44 [extremely small-fruited] and ZP65 [extremely large-fruited]) derived from a cross between Zaozhong No. 6 and Peluches were grown at the loquat germplasm resource preservation garden (South China Agricultural University, Guangzhou, China). Fruits of ZP44 and ZP65 were collected at 0, 7, 14, 28, 42, 56, 63, 77, 84, and 91 days past anthesis (DPA) with three biological replicates. Fruits collected at 0, 7, 28, 56, and 77 DPA were subjected to RNA-sequencing using the Illumina NovaSeq 6000 platform (150-bp paired-end reads). The RNA samples were extracted using the EASYspin Plus Plant RNA Extraction Kit (Aidlab, China).

DNA samples were extracted using young leaves and the M5 CTAB Plant gDNA Extraction kit (Mei5 Biotechnology Co., Ltd, Beijing, China). DNA samples of the F_1_ population and the two parental cultivars were subjected to whole-genome resequencing using the Illumina NovaSeq 6000 (150-bp paired-end) platform. DNA samples of germplasm accessions were subjected to whole-genome resequencing using the Illumina HiSeq 2000 (125-bp paired-end) platform.

### Mapping and variant calling

Clean reads generated from whole-genome resequencing were mapped to the “Seventh Star” reference genome [60], which was first released to the public at the initiation of this project in 2020, using BWA-MEM (BWA 0.7.11) [86]. Variants were called using GATK 4.2.0 [87] following the Best Practice pipeline (https://www.broadinstitute.org/gatk/guide/). The identified SNPs were filtered based on the following criteria: 1) bi-allelic SNPs; 2) quality >30; 3) Depth ≥ 4; 4) missing rate < 0.25; 5) minor allele frequency > 0.05.

### Linkage map construction and QTL identification

For linkage map construction, a Bin strategy (patent application number: 201810689069.8) developed by Beijing Biomarker Company (Beijing, China) was applied with HighMap software [61]. In brief, polymorphic SNPs with a homozygous genotype for Ninghaibai and Dafang were filtered out. The SNPs were first assigned to groups based on their physical locations on the reference genome, and linkage analysis was carried out for each pair of markers in each group. The linkage phases of markers were established based on recombination frequencies and mLOD values, which were further used to establish the linkage phases of parents. The genotypes of markers were corrected using the hidden Markov (HMM) model and Viterbi algorithm. Markers without recombination were partitioned into bins, which were used for linkage map construction using HighMap software. Only markers that were fully informative with phasing information available for both alleles for each parent were selected to construct the consensus map.

QTL mapping for fruit weight was carried out using the interval mapping model in MapQTL 6.0 software [88]. To determine the threshold of LOD scores, 1000 permutations were used. The LOD threshold was set at 6.2, corresponding to the 99.5% confidence interval. The collinearity between constructed linkage maps and physical locations was evaluated as previously described [61].

### Comparative genomic analysis of small-fruited and large-fruited loquat cultivars

To determine the genetic relationship of 20 commonly known small- or large-fruited loquat cultivars, a phylogenetic tree was constructed using the filtered SNPs and PHYLIP with 100 bootstraps [89]. Principal component analysis (PCA) was carried out using PLINK [90]. Genetic differentiation (*F*_st_) values (small-fruited vs large-fruited) were calculated using VCFtools [91] with the parameters “--fst-window-size 10000 --fst-window-step 5000”.

### RNA-seq analysis

Library construction and RNA-seq were performed by Wuhan Metware Biotechnology Co., Ltd. (Wuhan, China). HISAT2 [92] was used to map the clean reads generated from RNA-seq to the reference genome of “Jiefangzhong” [52], which became available to the public when this RNA-seq experiment was initiated in 2021. Novel transcript sequences were assembled using StringTie [93]. Annotation of novel transcripts was carried out by comparison against the KEGG, GO, NR, Swiss-Prot, trEMBL, and KOG databases. Principal component analysis was performed to determine the correlations among replicates. DEGs were identified using read counts in DESeq2 [94] with a threshold of |log_2_(Fold Change)| ≥ 1 and FDR < 0.05. To identify and compare gene expression patterns across different time points, the genes were clustered using the K-means method.

### Auxin metabolite profiling and analysis

Fruit tissues collected from ZP44 and ZP65 at the above five time points with three biological replicates were used for auxin metabolite profiling. Auxin metabolite extraction and quantification were performed using the AB Sciex QTRAP 6500 ultra-performance liquid chromatography–tandem mass spectrometry (LC–MS/MS) system following standard procedures. The procedures were performed as previously described [95], except that 1 mL methanol/water/formic acid (15:4:1, V/V/V) was used. Auxin contents were expressed as ng/g fresh weight based on the external standard method, with a series of standard solutions ranging from 0.01 ng/mL to 500 ng/mL, with the exceptions of TRP and SAG (0.2–10 000 ng/mL).

### Candidate gene search

To make use of the two available reference genomes for the candidate gene search, the coordinates of flanking markers/borders for identified QTL regions and top 1% *F*_st_ regions were extended from both directions, and sequences (500 bp in length) were extracted from the “Seventh Star” genome. The extracted sequences were mapped to the “Jiefangzhong” genome using Blastn to identify the corresponding genomic regions. To identify loquat homologs to known genes potentially associated with fruit weight, the keywords “cell division/elongation/enlargement/expansion/size”, “organ growth/size”, and “auxin signaling” were searched in the Swiss-Prot database. Related Arabidopsis genes were used to identify loquat homologs in both reference genomes using an All-Against-All Blast and OrthoMCL approach [96] (inflation value 1.5). Loquat homologs within the identified QTL or top 1% *F*_st_ regions or those that were DEGs were prioritized in the candidate gene search. These candidate genomic regions and genes were plotted on the two reference genomes using TBtools [97].

### qRT-PCR assays

Expression levels of the candidate genes were validated using quantitative real-time PCR. Primers were designed using Primer3 (https://primer3.ut.ee/). *Ejβ-actin* was used as the reference gene [98]. Primer sequences are provided in Table S14. Each biological replicate had three technical replications. A LightCycler 480 (Roche) and iTaq universal SYBR Green Supermix (Bio-Rad, USA) were used for the gene expression assays.

### Marker development and genotyping of *Eriobotrya* species

For the candidate SNP marker potentially associated with fruit weight, primers flanking the SNP were designed and used for PCR amplification with PrimeSTAR HS DNA Polymerase (TaKaRa, Japan). Sanger sequencing of PCR amplicons of the extremely small- and large-fruited loquat germplasm accessions and F_1_ offspring was performed by Sangon Biotech (Sangon Biotech, China). In addition, the primers were also used to genotype all known and available *Eriobotrya* species currently maintained in our loquat germplasm preservation garden (South China Agricultural University, Guangzhou, China). The Sanger results were visualized using DNA Baser v4 software (http://www.dnabaser.com/). Primers are provided in Table S14.

## Acknowledgements

This research was funded by the National Key R&D Program of China (2019YFD1000200), the Key-Area Research and Development Program of Guangdong Province (2018B020202011), Research Start-up Funding from South China Agricultural University, the National Natural Science Foundation of China (31901973), and the Collaborative Innovation Project from the People’s Government of Fujian Province & Chinese Academy of Agricultural Sciences (XTCXGC2021006).

## Author contributions

X.Y., W.S., and Z.P. designed and supervised the project. C.Z., Shuqing L., and Y.G. performed fruit weight phenotyping. J.C. and H.X. maintained the F_1_ population and helped with phenotyping. Z.L. and G.H. provided funding support. Shunquan L., X.Y., and X.C. secured loquat germplasm accession materials. Z.P. and C.Z. performed data analysis. Z.P. and C.Z. wrote the original manuscript draft. All authors reviewed and approved the manuscript.

## Data availability

All the raw sequencing data have been deposited at China National GeneBank (CNGB) database under Project accession number CNP0002296, Sample accession number CNS0462964-CNS0463140, and Experiment/Run accession number CNR0460669-CNR0460845.

## Conflict of interest statement

The authors declare that they have no conflict of interest.

## Supplementary data

Supplementary data is available at *Horticulture Research* online.

## Supplementary Material

Web_Material_uhac037
