# Supplementary figures and images for: Integration of genomics, transcriptomics and metabolomics identifies candidate loci underlying fruit weight in loquat
Source: Hortic Res. 2022 Feb 7;9:uhac037. doi: 10.1093/hr/uhac037 (PMC9071381; doi:10.1093/hr/uhac037)

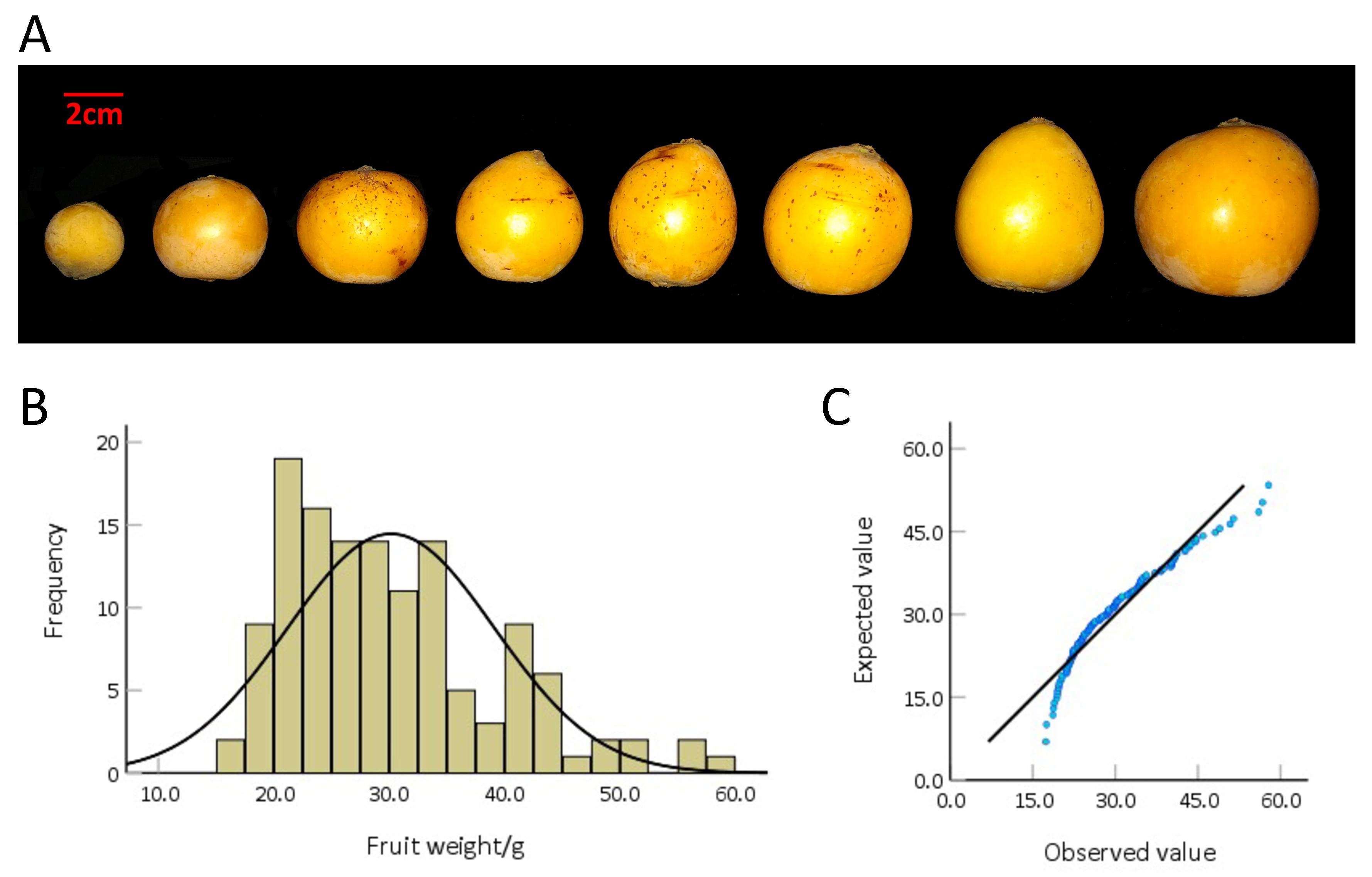

Supplement: Web_Material_uhac037 [file web_material_uhac037.zip › Figure S1.jpg]

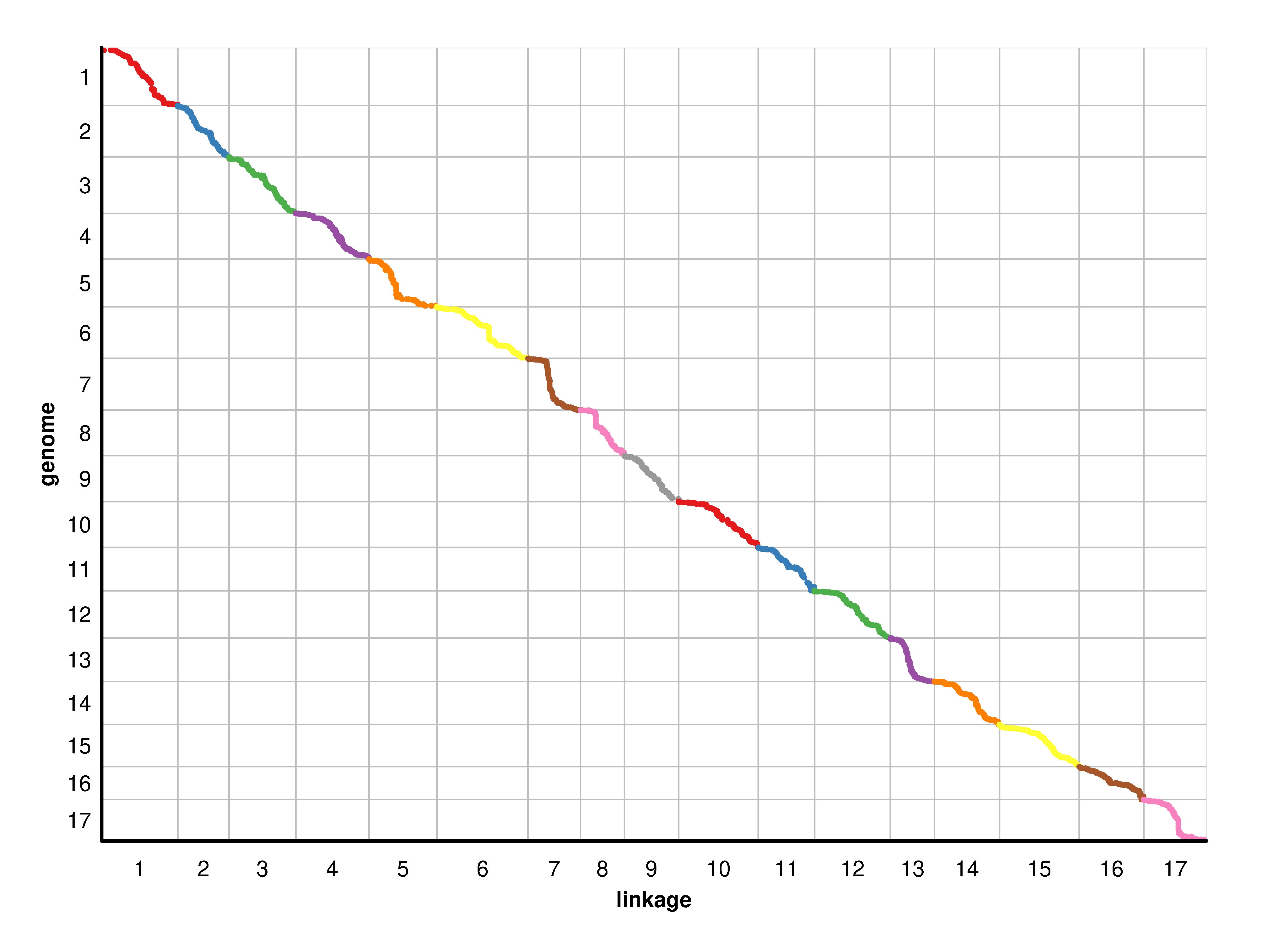

Supplement: Web_Material_uhac037 [file web_material_uhac037.zip › Figure S2.jpg]

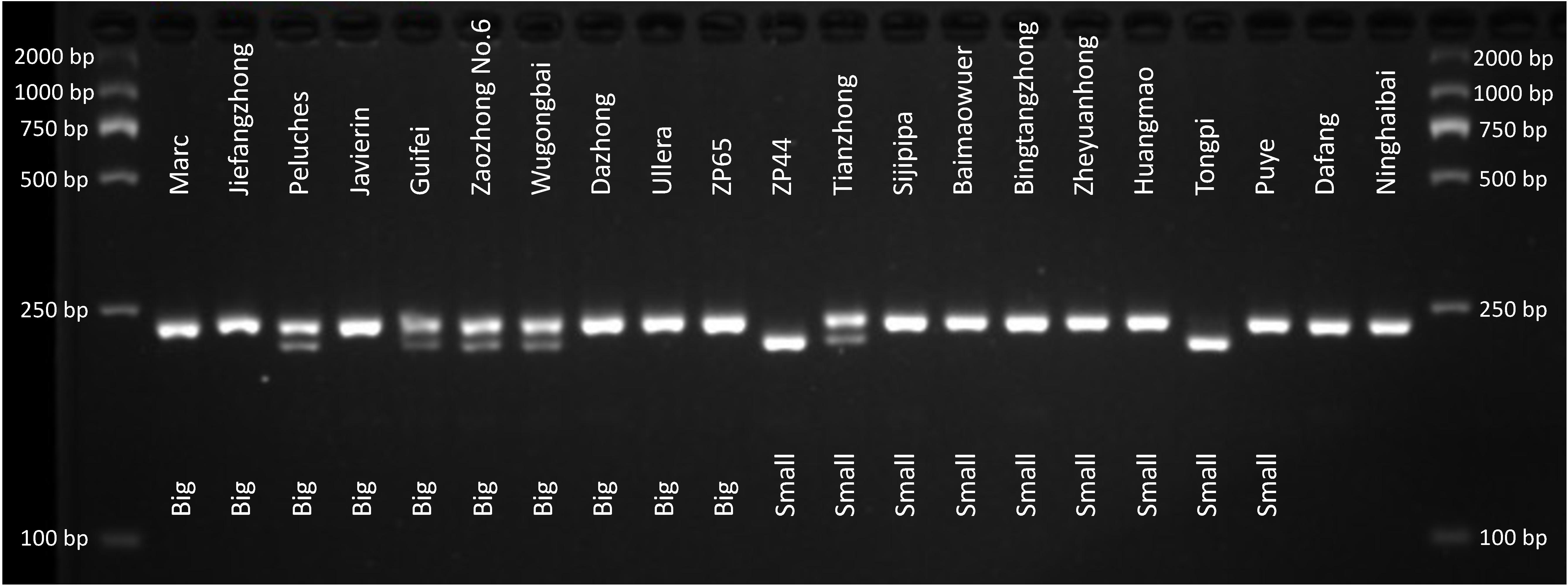

Supplement: Web_Material_uhac037 [file web_material_uhac037.zip › Figure S3-indel-revision.jpg]

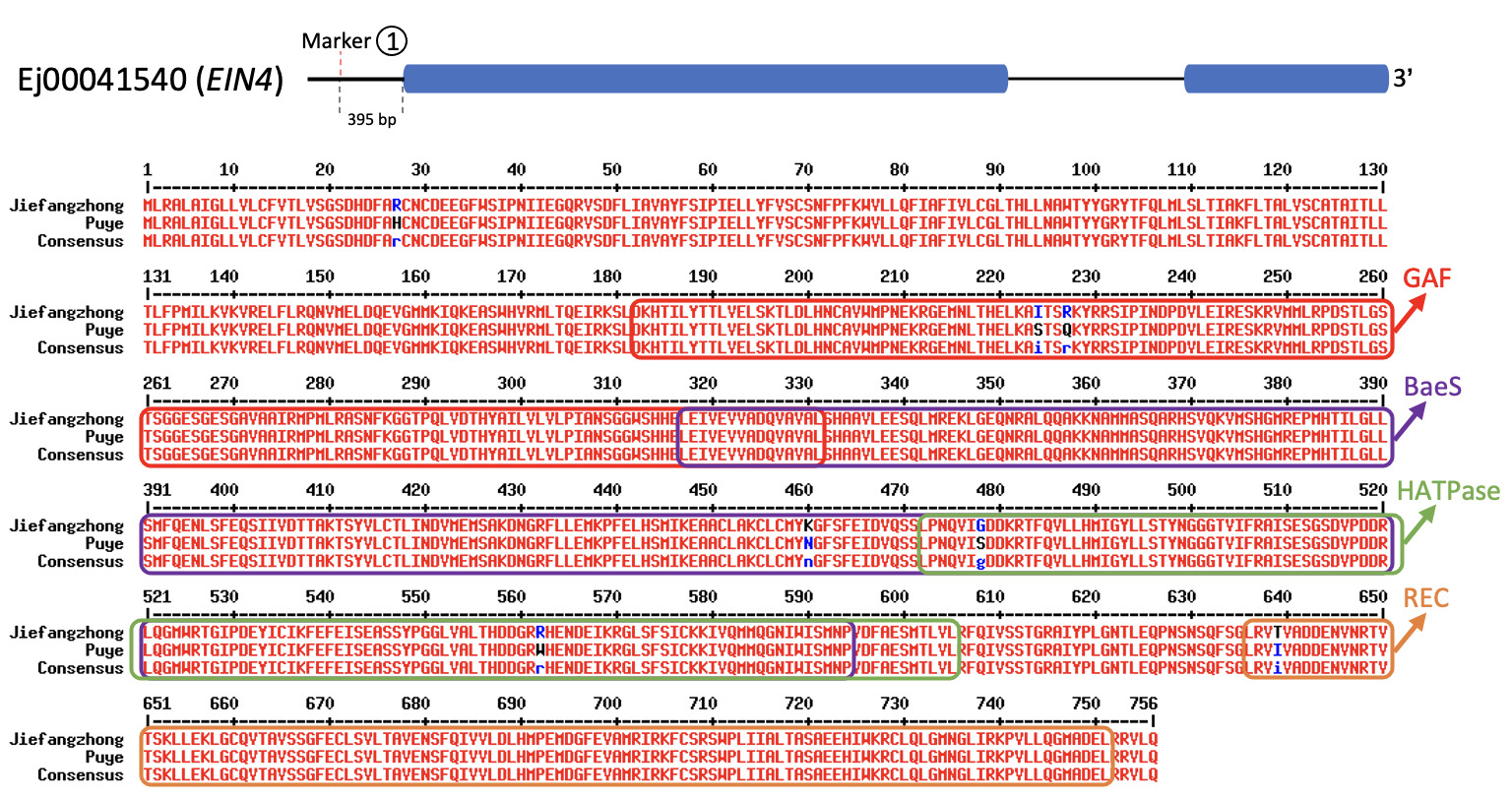

Supplement: Web_Material_uhac037 [file web_material_uhac037.zip › Figure S4-EIN4-revision.jpg]

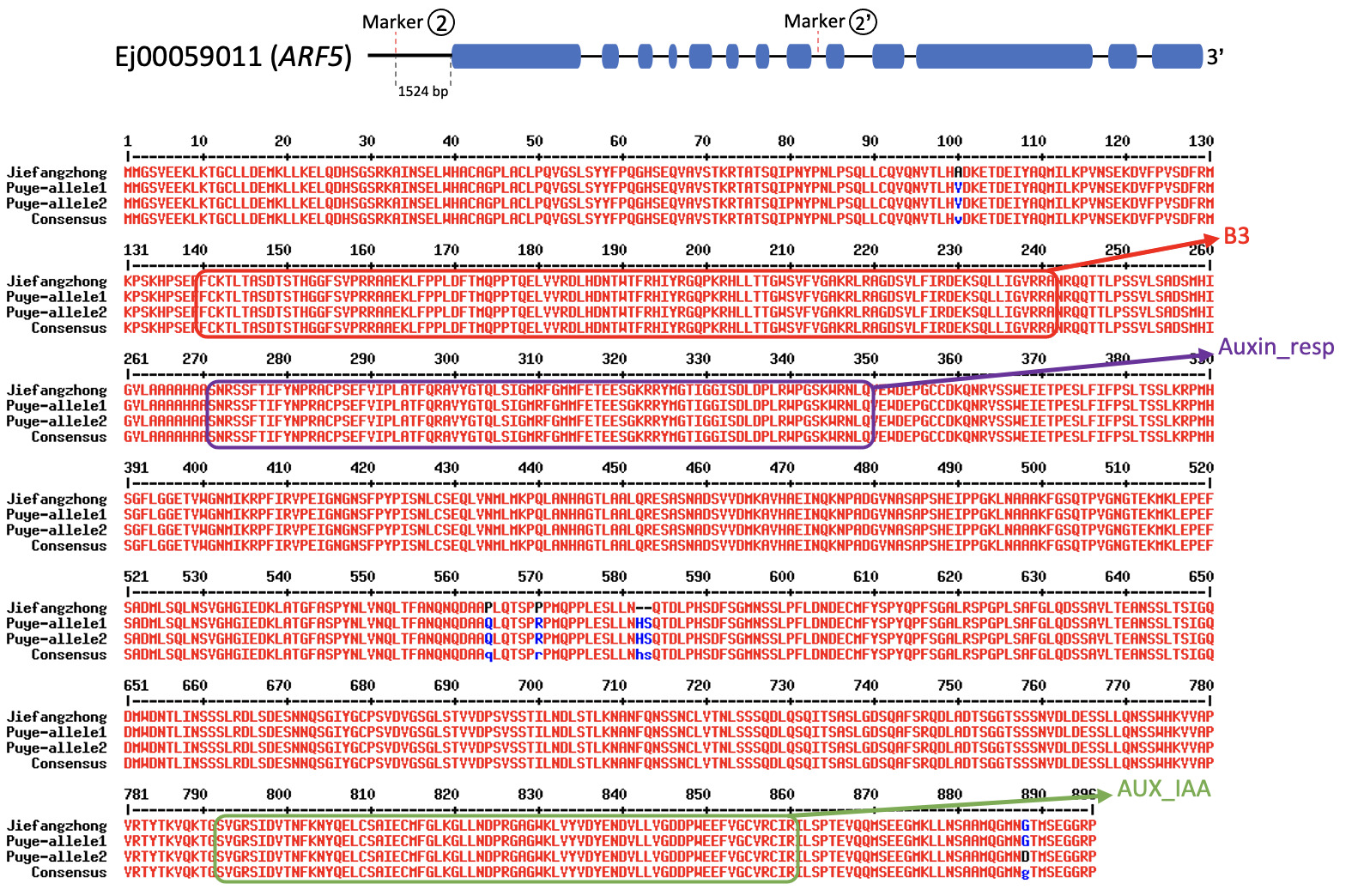

Supplement: Web_Material_uhac037 [file web_material_uhac037.zip › Figure S5-ARF5-revision.jpg]

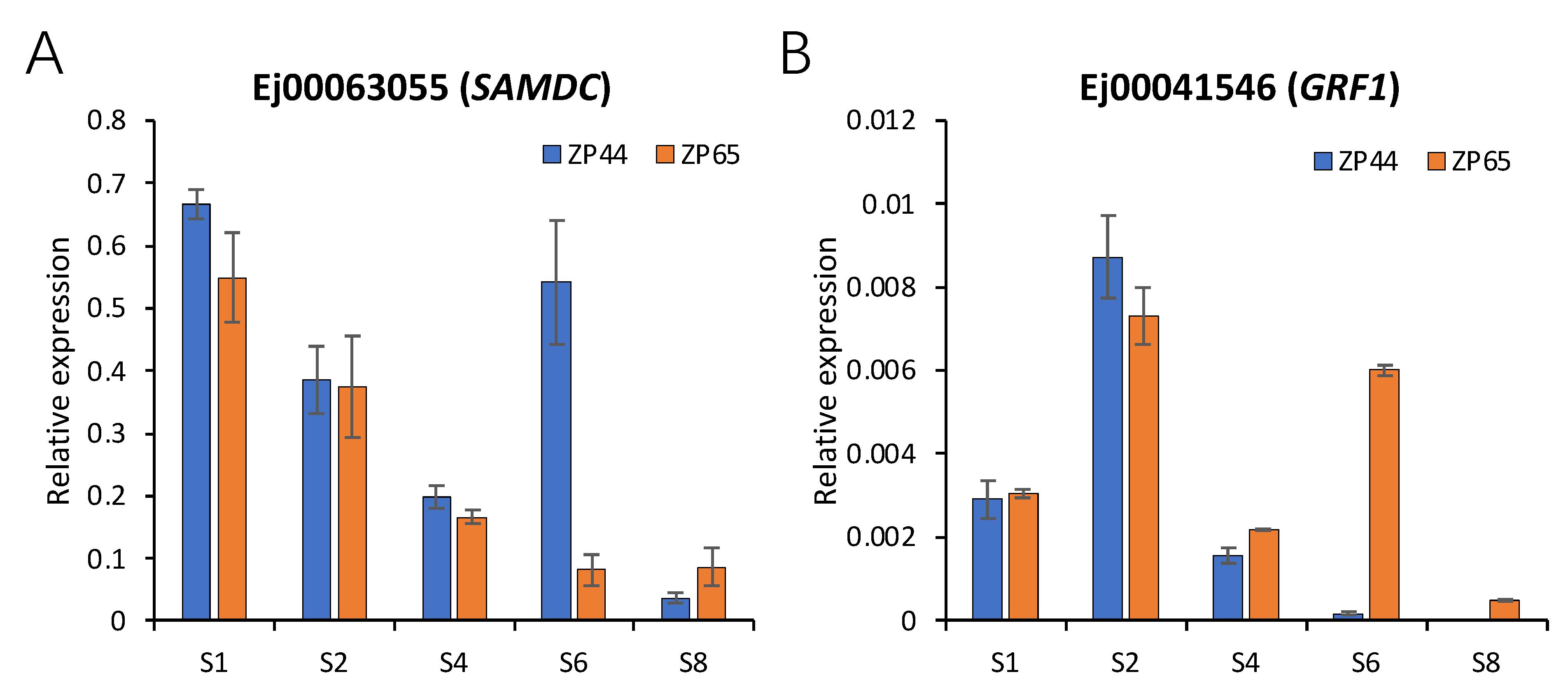

Supplement: Web_Material_uhac037 [file web_material_uhac037.zip › Figure S6-revision.jpg]
